# Thromboembolic stroke in patients with a HeartMate-II left ventricular assist device – the role of anticoagulation

**DOI:** 10.1186/s13019-015-0333-7

**Published:** 2015-10-15

**Authors:** Walter M. van den Bergh, Annemieke Oude Lansink-Hartgring, Abram L. van Duijn, Annemarie E. Engström, Jaap R. Lahpor, Arjen JC Slooter

**Affiliations:** 1 Department of Intensive Care, UMC Utrecht, Utrecht, The Netherlands; 2 Department of Critical Care, UMC Groningen, University of Groningen, PO BOX 30 001, Groningen, 9700 RB The Netherlands; 3 Department of Cardiothoracic Surgery, UMC Utrecht, Utrecht, The Netherlands; 4 Department of Cardiology, AMC, University of Amsterdam, Amsterdam, The Netherlands

**Keywords:** Heart-assist devices, Stroke, Anticoagulants, Coumarins, Platelet aggregation inhibitors

## Abstract

**Background and purpose:**

It is unknown what the optimal anticoagulant level is to prevent thromboembolic stroke in patients with left ventricular assist device (LVAD) support. We aimed to evaluate the relation between coagulation status and the occurrence of thromboembolic stroke in HeartMate-II LVAD assisted patients.

**Methods:**

Thirty-eight consecutive patients with a HeartMate-II LVAD were included. Coagulation status was classified according to INR and aPTT ratio at: 1) the moment of first thromboembolic stroke; and 2) during the two weeks preceding the first thromboembolic stroke to assess long-term coagulation status. In patients without stroke, coagulation status was determined just before heart transplant, VAD explantation or death, whichever came first, and at two weeks preceding these surrogate endpoints. Based on coagulation status, patients were divided in two groups: Group I (reference group) was defined as INR below 2 and aPTT ratio below 1.5; Group II (adequate anticoagulation) as INR above 2 or aPTT ratio above 1.5. Logistic regression analysis was performed to assess the odds ratio for developing stroke for patients with adequate anticoagulation compared to the reference Group.

**Results:**

Thromboembolic stroke occurred in six (16 %) patients, none within 2 weeks after LVAD implantation. Considering coagulation status at the time of event, patients in coagulation Group II had no decreased risk for thromboembolic stroke (OR 0.78; 95 % CI 0.12–5.0). Results for coagulation status 2 weeks prior of event could not be calculated as all six strokes occurred in Group II.

**Conclusion:**

In our experience anticoagulation within predefined targets is not associated with a reduced thromboembolic stroke risk in patients with a HeartMate-II LVAD on antiplatelet therapy. However, no firm statement about the effect of either anticoagulant or antiaggregant therapy can be made based on our study. A larger randomized study is needed to support the hypothesis that there may be no additional benefit of coumarin or heparin therapy compared with antiplatelet therapy alone.

## Background

Left ventricular assist devices (LVADs) have become an increasingly important therapeutic option in the treatment of end-stage heart failure. LVADs were initially introduced as ‘bridge-to-transplantation’. However, there is limited availability of donor organs and many patients with end-stage heart failure are not eligible for cardiac transplantation. Therefore, LVADs are applied as destination therapy as well, especially as outcomes for these patients have been demonstrated to be favorable when compared to medical therapy [1–4]. This results in more LVAD implantations, but also in a longer duration of implantation, which may increase the risk of complications including infections and stroke.

The reported incidence of stroke during LVAD support varies from 2 % to 47 % in studies evaluating different types of LVADs [5–8]. Anticoagulation is widely used for the prevention of thromboembolic stroke in LVAD patients, but the optimal therapeutic regime has been a matter of debate and no generally accepted guideline currently exists. Prophylactic administration of anticoagulants may enhance the risk of systemic bleeding and hemorrhagic stroke, which is already substantial due to LVAD-induced thrombopenia and acquired von Willebrand syndrome [9, 10]. It is therefore difficult to determine which anticoagulation regime is adequate.

The purpose of the current study was to evaluate the association between anticoagulation status and the occurrence of thromboembolic stroke in a single-centre cohort of patients treated with the HeartMate-II LVAD.

## Methods

### Study population and data sources

The study population consisted of all consecutive patients who underwent LVAD implantation in the Utrecht University Medical Center from 2006 until 2009. Only patients treated with the currently most frequently used continuous flow pump HeartMate-II LVAD were included in the analyses.

Demographic data, as well as data on the etiology of heart disease, the presence of risk factors for atherosclerosis, preoperative laboratory measurements and treatment characteristics were obtained through chart review. Long term outcome data were obtained through review of outpatient records. Data on the occurrence of cerebrovascular events were obtained through retrospective re-evaluation of imaging studies, in addition to review of clinical records and radiology reports.

In all cases, the intention of treatment with an LVAD was bridge-to-transplantation, therefore only patients who were eligible for cardiac transplant had LVAD implantation. All patients had a New York Heart Association (NYHA) classification IIIb or IV and received maximum medical treatment, including intravenous inotropic support. The decision for type of assist device depended on device availability and surgeon’s preference rather than on patient characteristics.

### Anticoagulation protocol

Patients in whom a HeartMate type-II was implanted received acetylsalicylic acid 100 mg daily and were additionally treated with heparin as soon as possible with the aim to keep the activated partial thromboplastin time (aPTT) ratio between 1.5–2.0, which mostly resulted in an aPTT of 50–70 s. When discharge from the ICU was within reach, usually between 1 and 2 weeks postoperatively, acenocoumarol was started with a target International Normalized Ratio (INR) for outpatients of 2.0–3.0. Heparin was discontinued once an INR of 2.5 had been reached. Acetylsalicylic acid 100 mg daily was continued in all cases. Low molecular weight heparin was not used during the study period.

### Outcomes and definitions

The primary outcome for the current analysis was the occurrence of thromboembolic stroke, including transient ischemic attack (TIA) or lacunar stroke. All cerebrovascular events were adjudicated by three authors (AvD, AS, WvdB) based on clinical information (occurrence, type of impairment, reversibility) and radiological investigations (area, size and aspect of infarction). In case of multiple events in one patient, only the first event was analyzed.

### Coagulation status

In patients with stroke, coagulation status was determined at the time of stroke and during the two weeks preceding the event. The latter analysis was considered important as the onset of clot development may well be begin days before the occurrence of a symptomatic embolus. In patients without stroke, coagulation status was determined just before heart transplant, VAD explantation or death, whichever came first, and at two weeks preceding these surrogate endpoints. In patients with on-going LVAD-support at the time of follow-up, coagulation status was based on the most recently recorded values and values recorded during the two weeks before.

Patients were divided in two groups based on a combined assessment of the INR and aPTT ratio. Group I (reference group) included patients with both INR and aPTT ratio below the target of 2.0 and 1.5, respectively. Group II (adequate anticoagulation) included patients with either INR above 2.0 or aPTT ratio above 1.5. Patients who did not receive heparin had no mandatory aPTT measurements. Hence, in case of missing aPTT values they apparently were not on heparin therapy and were attributed an aPTT ratio of 1.

### Statistical analysis

Continuous data were presented as median with interquartile range. Categorical data were presented as percentages. Differences between patients with different coagulation status were tested with the Fisher’s Exact test for categorical variables, and with the Mann-Whitney *U*-test for continuous variables that were not normally distributed. We imputed missing coagulation parameters using single imputation with the linear regression method with addition of a random error term. In 18 % of patients one or more values were missing, although not necessarily within two weeks preceding an event. The mean frequency of coagulation measurements within the two week preceding the event was 4.4 (STD 4.6). The occurrence of stroke was presented as both cumulative incidences (i.e. the percentage of patients with stroke for each category), and incidence rate (i.e. number of events per patient-years). Univariate logistic regression analysis was performed to assess the odds ratio for developing stroke for the two different categories of coagulation status at the time of the event. Group I was used as the reference category. All baseline characteristics were considered potential confounders [Table 1]. The influence of potential confounders was investigated by conducting multiple bivariate analyses. Any confounder that influenced the odds ratio for developing stroke by >10 % in the bivariate analyses was included in the multivariate model. Similar analyses were performed for mean coagulation status during the two weeks preceding the event. Kaplan-Meier curves for the two categories of coagulation status at the event were constructed. A *p* value < 0.05 (two sided) was considered to be statistically significant.

**Table 1:** Baseline characteristics according to coagulation status: Group I (low anticoagulation status) included patients with both INR below 2.0 and aPTT ratio below 1.50; Group II (adequate anticoagulation status) included patients with either INR ≥ 2.0 or aPTT ratio ≥ 1.50

	*Overall*	*Coagulation status* at the time of event			*Coagulation status* two weeks prior of event		
	(*n* = 38) median (Q1-Q3)	Group I (*n* = 12)	Group II (*n* = 26)	*p*	Group I (*n* = 8)	Group II (*n* = 30)	*p*
Age	45 (36–54)	46	44	*.94*	45	45	*.71*
Male sex (%)	25 (66 %)	7 (58 %)	18 (69 %)	*.71*	4 (50 %)	21 (70 %)	*.41*
Smoking (%)	24 (63 %)	7 (58 %)	17 (65 %)	*.73*	6 (75 %)	18 (60 %)	*.68*
Diabetes (%)	2 (5 %)	1 (8 %)	1 (4 %)	*.54*	0	2 (7 %)	*1.0*
Hypertension (%)	4 (11 %)	0	4 (15 %)	*.29*	0	4 (13 %)	*.56*
Hypercholesterolemia (%)	10 (26 %)	4 (33 %)	6 (23 %)	*.69*	3 (38 %)	7 (23 %)	*.41*
Prior MI (%)	5 (13 %)	1 (8 %)	4 (15 %)	*.99*	2 (25 %)	3 (10 %)	*.28*
Prior stroke (%)	4 (11 %)	2 (17 %)	2 (8 %)	*.58*	0	4 (13 %)	*.56*
Ischemic heart disease (%)	12 (32 %)	3 (25 %)	9 (35 %)	*.71*	5 (63 %)	7 (23 %)	*.08*
Hemoglobin	7.2 (6.5–7.9)	7.0	7.5	.*23*	6.4	7.4	*.10*
Leukocyte count	10.8 (8.3–13.9)	10.2	10.8	*.75*	8.5	10.8	*.18*
Platelet count	219 (164–296)	168	226	*.34*	192	226	*.77*
Creatinin	117 (96–150)	103	120	*.11*	119	114	*.54*
Blood urea nitrogen	10.6 (7.4–15.3)	90	11.8	*.15*	9.2	11.8	*.31*
ASAT	66 (32–192)	42	77	*.24*	126	62	*.99*
ALAT	71 (38–216)	53	77	*.31*	64	71	*.57*
LDH	481 (297–726)	354	542	*.04*	465	504	*.68*
Cardiac index	1.78 (1.50–2.01)	1.81	1.70	*.77*	1.86	1.69	*.45*
IABP treatment (%)	4 (11 %)	2 (17 %)	2 (8 %)	*.58*	1 (13 %)	3 (10 %)	*.99*
Temporary VAD (%)	4 (11 %)	2 (17 %)	2 (8 %)	*.58*	2 (25 %)	2 (7 %)	*.19*
Concomitant RVAD	0	0	0	*-*	0	0	*-*

### Ethics

The study complies with the Declaration of Helsinki and good clinical practice guidelines. We obtained ethics committee approval from the University Medical Center Utrecht. Written informed consent was waived by the ethics committee. Patient information was anonymized and de-identified prior to analysis.

## Results

### Patients

From 2006 through 2009 a total of 41 consecutive patients underwent a HeartMate-II LVAD implantation in our hospital. Three patients were excluded from the primary analysis on thromboembolic stroke; one suffered hemorrhagic stroke (aPTT ratio at that time was 2.60), one post-anoxic stroke, and one symptomatic MELAS (mitochondrial myopathy, encephalopathy, lactate acidosis and stroke like episodes). Tables 1 and 2 show the baseline characteristics and clinical course data for the remaining 38 patients. Over one-fifth of patients required preoperative mechanical circulatory support with an intra-aortic balloon pump or a temporary VAD (e.g. Impella percutaneous left ventricular assist device). Median duration of support was 279 days (IQR 134 – 540); median duration of follow-up was 275 days (IQR 75 – 522). No patients underwent concomitant RVAD-implantation. Complication rate for tamponade, rethoracothomy and gastro-intestinal bleeding was higher in Group II, but due to low numbers didn’t reach statistical significance. Half of these complications occurred in the first 48 h after implantation, and two-third occurred within the first week.

**Table 2:** Clinical course and outcome according to coagulation status: Group I (low anticoagulation status) included patients with both INR below 2.0 and aPTT ratio below 1.50; Group II (adequate anticoagulation status) included patients with either INR ≥ 2.0 or aPTT ratio ≥ 1.50

	*Overall* (*n* = 38)	*Coagulation status* at the time of event			*Coagulation status* two weeks prior of event		
	*N* (%)	Group I (*n* = 12)	Group II (*n* = 26)	*p*	Group I (*n* = 8)	Group II (*n* = 30)	*p*
Complications							
Tamponade	6 (16 %)	0	6 (23 %)	*.15*	1 (13 %)	5 (17 %)	*.99*
Rethoracothomy	7 (18 %)	0	7 (27 %)	*.07*	1 (13 %)	6 (20 %)	*.99*
Infection	9 (24 %)	2 (17 %)	7 (27 %)	*.69*	2 (25 %)	7 (23 %)	*.99*
GI bleeding	2 (5 %)	0	2 (8 %)	*.99*	0	2 (7 %)	*.99*
RV failure	8 (21 %)	2 (17 %)	6 (23 %)	*.99*	2 (25 %)	6 (20 %)	*.99*
AKI	2 (5 %)	1 (8 %)	1 (4 %)	*.54*	1 (13 %)	1 (3 %)	*.38*
Median duration of follow-up in days (Q1-Q3)	275 (75–522)	299	242	*.94*	314	*254*	*.96*
Median duration of LVAD support in days (Q1-Q3)	279 (134–540)	321	279	*.96*	314	279	*.97*
Death before transplant	4 (11 %)	1 (8 %)	3 (12 %)	*.99*	1 (13 %)	3 (10 %)	*.99*
Transplanted and alive	9 (24 %)	2 (17 %)	7 (27 %)	*.69*	1 (13 %)	8 (27 %)	*.65*
Transplanted and deceased	1 (3 %)	0	1 (4 %)	*.99*	0	1 (3 %)	*.99*
Weaned from LVAD	3 (8 %)	0	3 (12 %)	*.54*	1 (13 %)	2 (7 %)	*.99*
On-going LVAD support	21 (55 %)	9 (75 %)	12 (46 %)	*.16*	5 (63 %)	16 (53 %)	*.71*
Thromboembolic stroke	6 (16 %)	2 (17 %)	4 (15 %)	*.99*	0 (0 %)	6 (20 %)	*<.001*
Stroke rate during LVAD support (event/patient/year)	.160	.169	.156	*.99*	-	.173	*<.001*

### Coagulation status

At the time of the primary or surrogate event, the reference Group I consisted of 12 patients and Group II consisted of 26 patients. When calculating mean coagulation during the two weeks preceding the event, 8 patients fell into Group I, and Group II consisted of 30 patients. The duration of sub-therapeutic anticoagulation range from several hours to one week. No differences were demonstrated between these groups with regard to baseline characteristics, in particular in pre-LVAD right ventricular failure, which parameter was previous associated with stroke development [11].

### Thromboembolic stroke

**Fig. 1:**
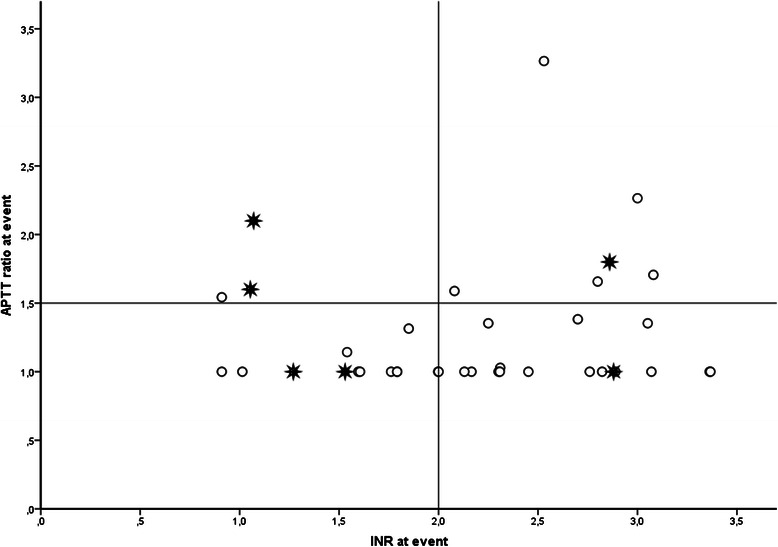
Thromboembolic stroke incidence according to coagulation status at event. Patients within Group I are positioned in the square in the lower left hand corner; Group II consists of the 3 squares that enclose Group I. O = no thromboembolic stroke. * = thromboembolic stroke

Overall, 6 thromboembolic strokes occurred in 6 patients (16 %). All occurred after 14 days after LVAD implantation or later. Median time of stroke occurrence was 43 days (IQR 14–440) after LVAD implantation. In general, more strokes occurred in Group II compared with the reference Group I [Table 2] [Fig. 1].

**Fig. 2:**
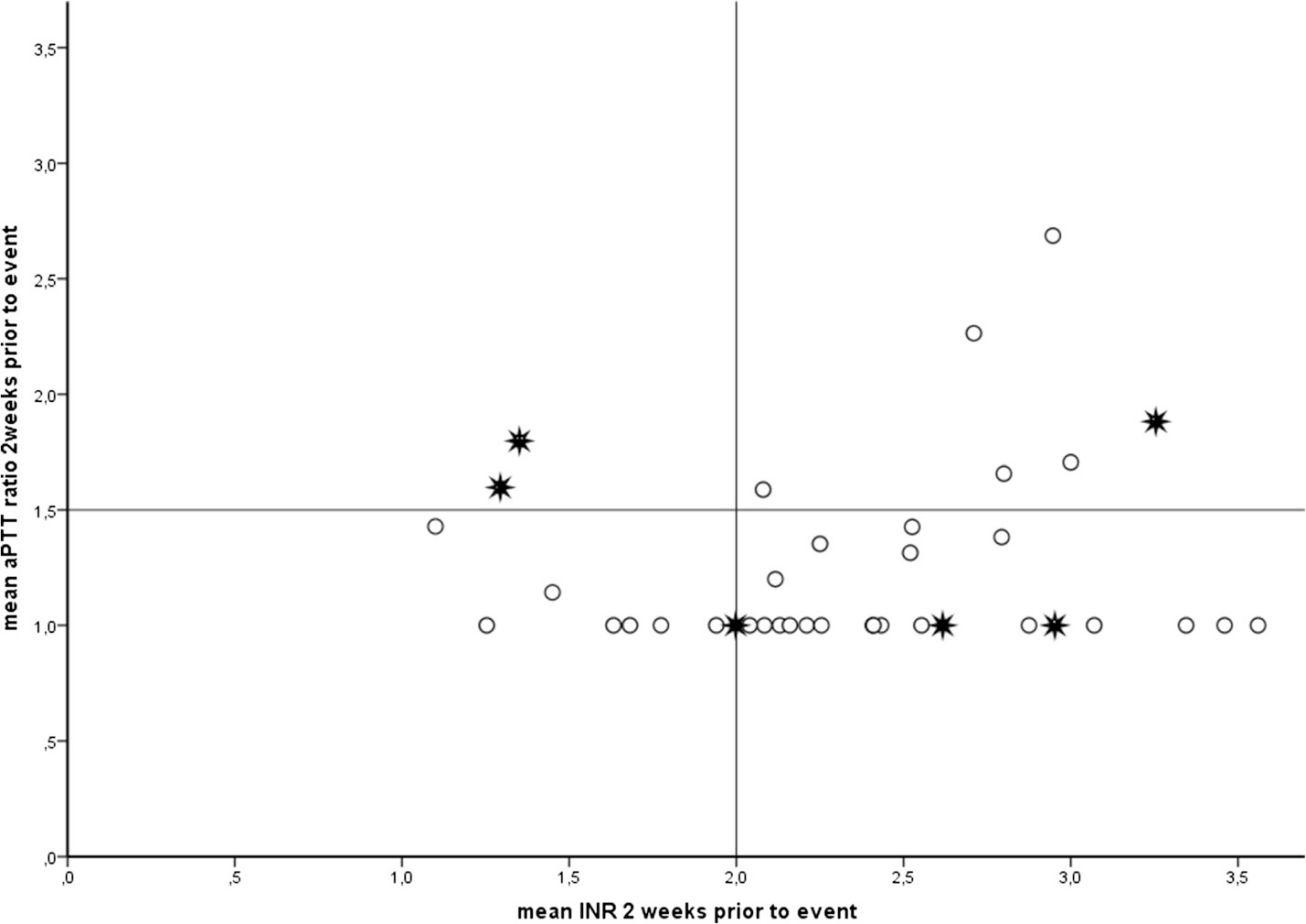
Thromboembolic stroke incidence according to coagulation status 2 weeks prior to event. Patients within Group I are positioned in the square in the lower left hand corner; Group II consists of the 3 squares that enclose Group I. O = no thromboembolic stroke.  = thromboembolic stroke

**Fig. 3:**
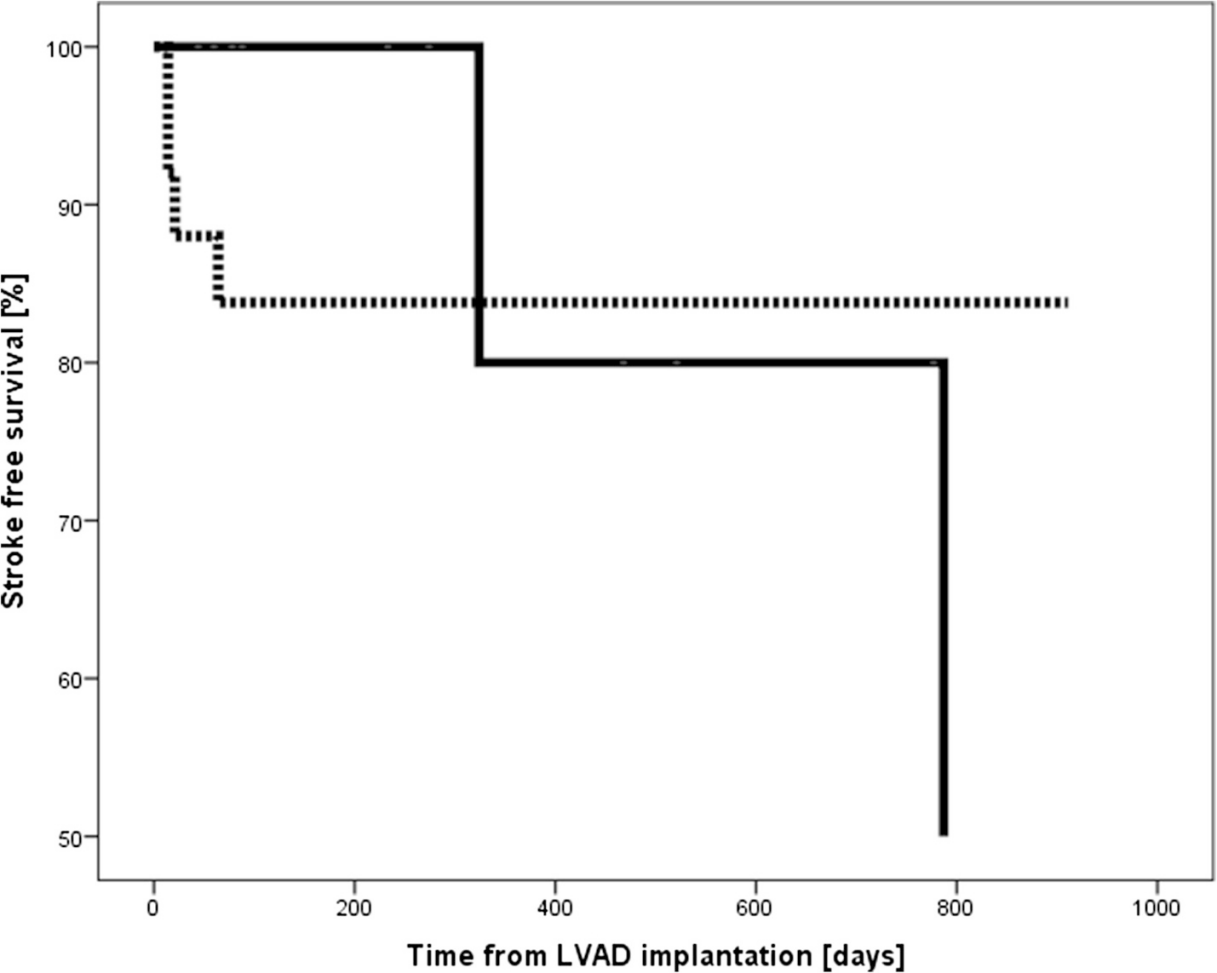
Kaplan-Meier survival curves showing stroke free survival according to coagulation status at event (Fig. 3). ______ Group I ………. Group II

**Fig. 4:**
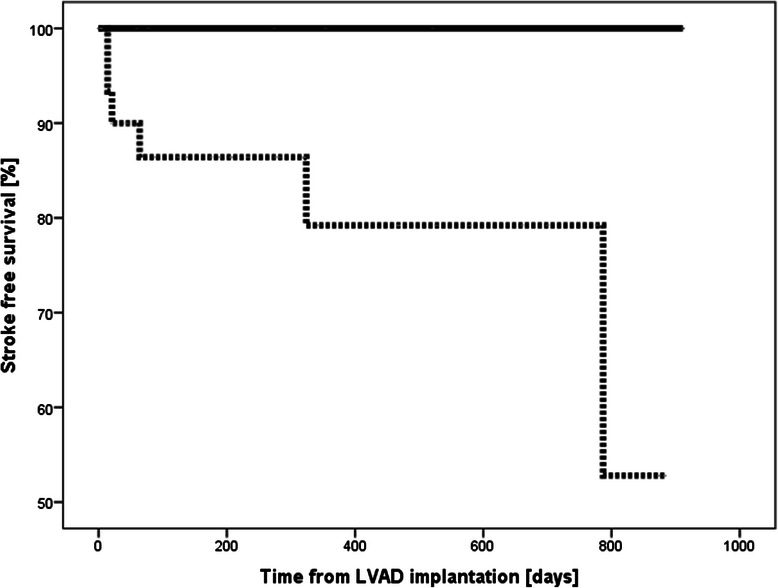
Kaplan-Meier survival curves showing stroke free survival according to coagulation status 2 weeks prior to event (Fig. 4). ______ Group I ………. Group II

Considering the analysis for coagulation status at the time of stroke, the Kaplan-Meier estimates for 1-year stroke free survival were 100 % for patients in Group I and 84 % for patients in Group II [Fig. 2]. When coagulation status was based on the two weeks preceding the event the Kaplan-Meier estimates for 1-year stroke free survival were 100 % for patients in Group I and 86 % in Group II [Figs. 3 and 4].

Compared with reference Group I, the adjusted odds ratio for the development of thromboembolic stroke was 0.53 (95 % CI 0.06–4.6) for coagulation Group II [Table 3]. When coagulation status was based on the two weeks preceding the event, the odds ratio could not be calculated as all strokes occurred in patients with adequate anticoagulation (Group II).

**Table 3:** Influence of coagulation status on the occurrence of thromboembolic stroke – results of logistic regression analyses

	Crude odds ratio (95 % CI)	Adjusted odds ratio (95 % CI)
*At the time of event:*		
Coagulation status I	- (reference)	-
Coagulation status II	0.91 (0.14 – 5.8)	0.63 (0.07 – 5.5)^*a*^
*Two weeks prior of event:*		
Coagulation status I	- (reference)	-
Coagulation status II	N/A^b^	N/A^b^

Results are presented as odds ratios with 95 % confidence intervals (CI)

^*a*^Adjusted for age, history of hypertension, history of hypercholesterolemia, ischemic heart disease

^*b*^Could not be calculated as all strokes occurred in patients assigned to Group II

## Discussion

The main finding from our study is that patients assisted by a HeartMate-II LVAD with an INR or aPTT above predefined targets did not have a reduced stroke incidence. Another important finding was that there was a trend towards more early complications, e.g. rethoracothomy and gastrointestinal bleeding, in patients with adequate anticoagulation.

It is widely assumed that the optimal INR to minimize the risk of both thromboembolic and hemorrhagic events needs to be between 1.5 and 2.5, in addition to the use of aspirin. A study in out-patients showed that the risk of thrombotic events increased when the INR was below 1.5, whereas the risk of hemorrhagic events sharply increased with an INR above 2.5 [6]. One important limitation of that study is that INR levels were recorded only at monthly intervals and at the time of a clinical event, whereas INRs for outpatients can change widely and over much shorter time periods according to patient conditions. Furthermore, patients were not included in the study until they were successfully discharged. Thus, patients who had peri-operative bleeding or thromboembolic events before discharge that was not related to the degree of oral anticoagulation were excluded. Also, numbers were very low within this sub-study in out-patients only and no statistics were performed to show that the numbers were statistically significant [6]. Several other studies also found that suboptimal anticoagulation was associated with neurological events, but it all concerns observational studies with low absolute numbers and several other risk factors were identified [12, 13]. Another limitation is that a single measurement below the target value, often at the moment stroke occurred, was used for analysis. Moreover, hemorrhagic stroke was associated with increased coagulation status which is the reason for some to advocate a mild anticoagulation protocol [14].

A limitation of our study is its retrospective design, withholding an increased risk of missing values. To overcome this problem several measures were taken. Firstly, the INR was, when appropriate, calculated from the PT ratio and the aPTT ratio from the heparin ratio. However, although the mentioned tests are related, each marker is calculated using different types of reagent and is therefore not freely interchangeable. The results of these calculations must therefore be seen as estimates. Secondly, our hospital protocol commands aPTT (or heparin ratio) being measured 4 times daily when heparin is administered. As a consequence, if an aPTT or heparin ratio value was unknown, it was presumed that the patient had no heparin therapy at that time and an aPTT ratio of 1 was filled in. However, in outpatients on coumarin, INR measurements are performed with larger intervals and therefore a missing value at a certain moment does not exclude coumarin therapy. To overcome this, an imputation procedure was performed to fill in the remaining blanks. Data omission rarely occurs at random, but is often related to other observed patient characteristics. Excluding subjects with missing values not only leads to loss of statistical power, but also to biased results [15–17]. To decrease bias and increase statistical efficiency, we therefore chose to impute missing values, rather than to perform complete-case analyses.

Another possible limitation is that although many risk factors were assessed, we didn’t systematically collect pre-implant atrial fibrillation or the CHA2DS2-Vasc score in our patients, which is associated with a higher risk of thromboembolic events in patients with continuous flow LVAD [18]. None of the 6 stroke patients had atrial fibrillation at the time of stroke, so the impact on our results is limited.

Throughout the study period various models of LVAD have been used and results of this study may not be translated to the other used devices. We chose to restrict the analysis to one particular LVAD type (i.e. Heart Mate II) to rule out that the observed differences in thromboembolic events were due to the different type of LVAD used.

The three patients with primary intracranial hemorrhages, global ischemia and MELAS, were left out of the case control analyses, because these events are not related to thromboembolism. The low incidence of intracranial hemorrhage, however, may indicate that anticoagulation with coumarin or heparin does not lead to an increased risk of intracranial hemorrhage. It is well known that dual or triple therapy increase the risk of bleeding complications [19]. This is further illustrated by the higher incidence of tamponade and rethoracothomy in the group of patients with adequate anticoagulation status.

To our knowledge, no randomized clinical trial on anticoagulation in patients with VAD, has ever been performed. We have previously performed a systematic review on the relation between anticoagulation protocols and occurrence of stroke after LVAD implantation, in which we found that platelet aggregation inhibitors are the most important in preventing thromboembolic stroke and should be part of the anticoagulation protocol [20]. The World’s Longest-Supported HeartWare Ventricular Assist Device Patient has been managed on low-dose aspirin (100 mg three times per week) without thrombotic complications [21].

Based on the results of our study it is not clear if postoperative heparin decrease the number of thromboembolic strokes in patients with a HeartMate-II support. Since heparin therapy may increase the risk of short-term systemic bleeding complications these findings indicate that the postoperative use of heparin after HeartMate II implantation may be refrained or postponed in selected patients with an anticipated increased bleeding risk.

Although not undoubtedly effective in preventing long term stroke in our study, the recently reported multicenter increase in the incidence of pump thrombosis with the HeartMate II LVAD may be an additional reason apart from patient characteristics to maintain anticoagulation with coumarins [22]. Both in-patient and device complication remains a challenge for bioengineering, which advances may be even more important than anticoagulants regimes as preclinical studies in LVAD technology demonstrates that design changes may offer a more robust alternative to antiplatelet drugs [23]. Within the study population, no LVADs needed to be replaced, so probably there was no occurrence of (significant) pump thrombosis, but unfortunately we didn’t systematically assessed this endpoint.

The suggestion from the results of our study that antiplatelet therapy could be sufficient without the addition of coumarins should be supported by larger studies, and more important by a qualitative assessment of the coagulation status such as thromboelastometry and platelet function assays besides the classical parameters INR, PTT, and platelet count. With such information, it would be possible to calibrate the antithrombotic therapy, deciding dose variation or withdrawal of either anticoagulant or antiaggregant therapy, on a mechanistic basis.

## Conclusion

Thromboembolic stroke remains a feared complication in patients assisted by a HeartMate-II LVAD. A vast majority of strokes occurred despite adequate anticoagulation according to the protocol of our institute. Methodological shortcomings of our study prevent a firm statement on the effect of coumarin or heparin in addition to antiplatelet therapy. It is currently unclear how stroke can be prevented in this patient category. A randomized clinical trial is warranted in order to improve outcome of this frail and growing patient category.

## Abbreviations

aPTT: Activated partial thromboplastin time
INR: International Normalized Ratio
LVAD: Left ventricular assist device
OR: Odds ratio
PTT: Partial thromboplastin time
MELAS: Mitochondrial myopathy, encephalopathy, lactate acidosis and stroke like episodes
NYHA: New York Heart Association
RVAD: Right ventricular assist device
TIA: Transient ischemic attack
VAD: Ventricular assist device
